# Obstinate Hiccough Cured by Muriate of Pilocarpine

**Published:** 1880-07

**Authors:** 


### OBSTINATE HICCOUGH CURED BY MURIATE OF PILOCARPINE.

Dr. Ortille reports: "As the singultus persisted even during the sleep produced by morphine injections, and the strength of the patient was becoming greatly reduced, a hypodermic injection of half a grain of pilocarpine was at last administered. This produced abundant perspiration and salivation, and the hiccough ceased at once.—*Atl. Med. Cent. Zeit.; Cinc. Med. News.*

